# Management of Chronic Disseminated Intravascular Coagulation Associated with Aortic Aneurysm/Dissection

**DOI:** 10.1155/2019/6204652

**Published:** 2019-04-02

**Authors:** Shigeru Koba, Tomoya Yamaguchi, Kenji Miki, Hiroshi Makihara, Shinsaku Imashuku

**Affiliations:** ^1^Department of Internal Medicine, Uji-Tokushukai Medical Center, Uji 611-0041, Japan; ^2^Department of Laboratory Medicine, Uji-Tokushukai Medical Center, Uji 611-0041, Japan

## Abstract

Disseminated intravascular coagulation (DIC) is a systemic life-threatening process that can cause thrombosis and hemorrhage. Chronic DIC has been associated with aortic aneurysm/dissection. Aortic aneurysm/dissection should be included in the differential diagnosis of elderly patients with hemorrhagic diathesis due to DIC of uncertain etiology. Treatment depends on various factors, including the severity of underlying disease, extent of DIC, and patient comorbidities, as well as the ability of the patient to maintain activities of daily living once discharged from the hospital. This report describes the clinical characteristics of four elderly patients with chronic DIC associated with aortic aneurysm/dissection who were treated in our institution. We also offer the recommendations around most appropriate nonsurgical treatment of these patients.

## 1. Introduction

Disseminated intravascular coagulation (DIC) is an acquired disorder that occurs in patients with a wide variety of clinical conditions, including leukemia, other cancers, and sepsis. Patients with DIC also experience secondary fibrinolysis, with the degree of activation of the fibrinolytic system dependent on the underlying disease [1, 2]. Chronic DIC is a rare but life-threatening complication of aortic aneurysm/dissection [3, 4], which was first described in 1967 [5]. However, compensatory mechanisms in aortic chronic DIC may result in mild or even occult clinical symptoms [6, 7], leading to a delayed diagnosis. Case reports have described various treatments in patients with chronic DIC associated with aortic aneurysm/dissection [3,4,8–15], but the optimal type of treatment has not yet been determined.

Two types of treatment are available for patients with chronic aortic DIC: surgery or nonsurgical treatment by using systemic anticoagulation. Although open or endovascular surgery can result in a radical cure, there is a risk of bleeding; moreover, surgical methods are not suitable for some patients, depending on their age, health conditions, and disease backgrounds [3, 4]. Three major nonsurgical strategies are available for patients with aortic DIC: anticoagulation therapy [8–10], antifibrinolytic therapy [11], and a combination of both [12]. The main purpose of these treatments is to halt the cycle of thrombus formation and destruction, by blocking the consumption of systemic coagulation factors, fibrinogen and platelets and by halting further bleeding. This report describes the clinical course, treatment, and outcomes of four elderly patients with chronic DIC accompanied with aortic aneurysm/dissection, all of whom developed excessive fibrinolytic activity.

### 1.1. Case Reports

The summary of the four patients is given in Table 1.

**Figure 1:**
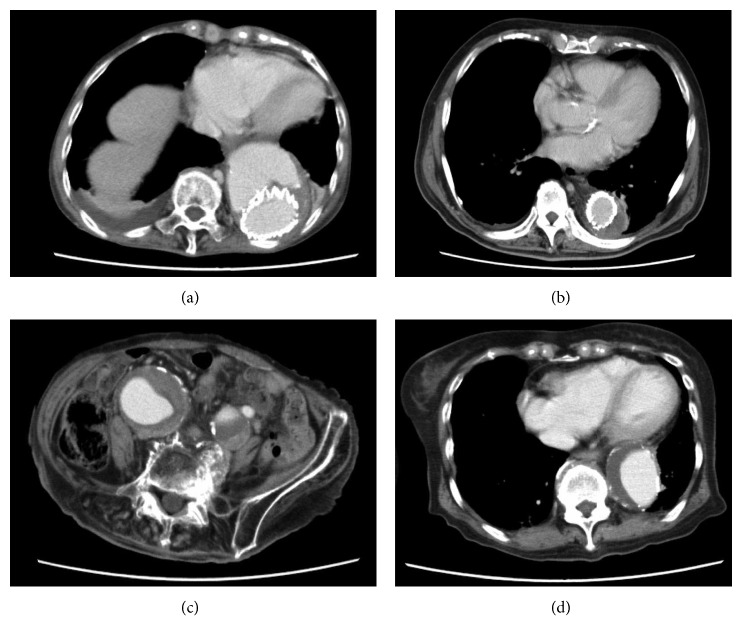
Contrast-enhanced axial CT findings showing aortic aneurysm/dissection in (a) Patient 1, showing a stent graft in the descending aorta; (b) Patient 2, showing a stent graft in the descending aorta; (c) Patient 3, showing aneurysms in the bilateral common and internal iliac arteries; and (d) Patient 4, showing an aneurysm in the descending aorta.

**Figure 2:**
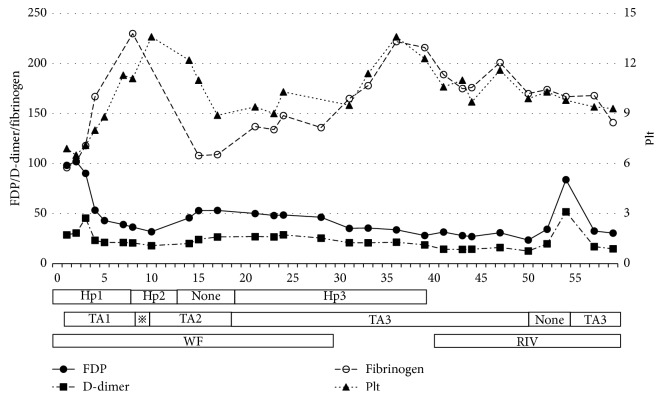
Clinical course correlation of treatment and improvement of DIC-related data in Patient 1. (Vertical axis) FDP = fibrin degradation product (*µ*g/ml); D-dimer (*µ*g/ml); fibrinogen (mg/dl); Plt = platelet count (x10e4/*µ*l). (Horizontal axis) days; Hp = heparin: Hp1 = continuous intravenous infusion (10,000 U/day), Hp2 = continuous intravenous infusion (8,000 U/day), and Hp3 = subcutaneous administration (5,000 U twice daily); TA = tranexamic acid: TA1 = intravenous infusion (250 mg twice daily), 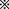 = intravenous TA (250 mg twice daily) + oral TA (750 mg/day), TA2 = oral (750 mg/day), and TA3 = oral (1500 mg/day); WF = warfarin oral; RIV = rivaroxaban oral (15 mg/day).

Patient 1 was an 84-year-old woman who had undergone thoracic endovascular aortic repair (TEVAR) for an aortic dissected aneurysm (Figure 1(a)) and was hospitalized for thrombocytopenia and abnormal coagulation. Her laboratory data showed hemoglobin (Hb) 7.9 (reference; 11–16) g/dl, platelet count 79,000 (reference; 150,000–360,000)/*μ*l, fibrinogen degradation product (FDP) of 101.5 (reference; <5) *μ*g/ml, D-dimer of 49.8 (reference; <1.0) *μ*g/ml, fibrinogen 98 (reference; 200–400) mg/dl, thrombin-antithrombin complex (TAT) 40.5 (reference; <3) ng/ml, and plasmin-*α*2 plasmin inhibitor complex (PIC) 12.7 (reference; <0.8) *μ*g/ml. Prior to admission, she had been treated with warfarin. Our vascular surgeons regarded her aneurysm condition after TEVAR as inoperable. She was started on a continuous intravenous infusion of 10,000 units/day heparin and 250 mg twice daily intravenous tranexamic acid. This combination treatment was effective as her plasma FDP and D-dimer concentrations decreased while her fibrinogen level and platelet count increased (Figure 2). To facilitate her discharge, she was switched from intravenous to oral tranexamic acid (750 mg/day) and from intravenous to subcutaneous administration of heparin calcium (5,000 units twice daily), and warfarin was stopped. Although this combination was effective, the patient was intolerant of subcutaneous heparin calcium because of pain, and she was transitioned to oral rivaroxaban 15 mg/day for discharge from hospital.

**Figure 3:**
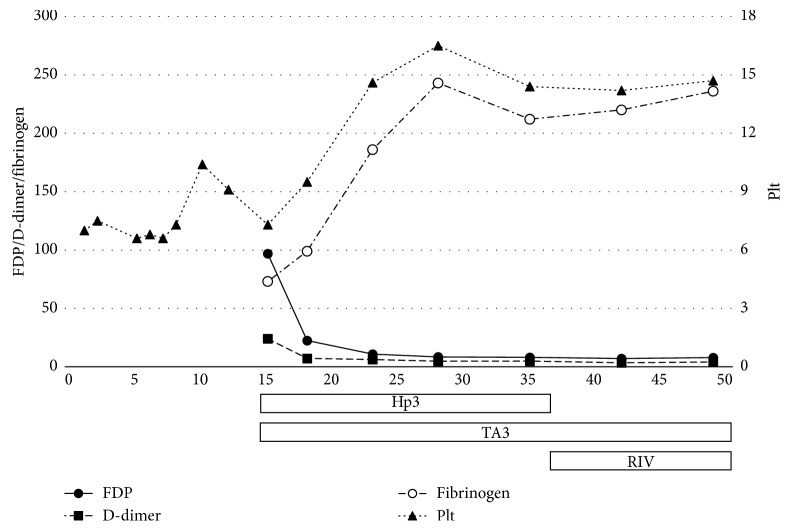
Clinical course correlation of treatment and improvement of DIC-related data in Patient 2. (Vertical axis) FDP = fibrin degradation product (*µ*g/ml); D-dimer (*µ*g/ml); fibrinogen (mg/dl); Plt = platelet count (x10e4/*µ*l). (Horizontal axis) days; Hp = heparin: Hp3 = subcutaneous administration (5,000 U twice daily); TA3 = oral (1500 mg/day); RIV = rivaroxaban oral (15 mg/day).

Patient 2 was an 87-year-old man who had undergone TEVAR for a Stanford type B aortic dissection 7 months earlier (Figure 1(b)). Thereafter, he was sent to a rehabilitation center, where his plasma FDP and D-dimer increased gradually, while fibrinogen and platelet count decreased, and anemia progressed. He received transfusions of packed red blood cell (PRBC) and platelet concentrate (PC) several times (precise units unknown), although the cause of his abnormal coagulopathy was not adequately assessed. Following persistent gingival bleeding for 2 weeks, he was transferred to our hospital for evaluation. His laboratory data showed Hb 7.9 g/dl, platelet count 73,000/*μ*l, FDP 96.8 *μ*g/ml, D-dimer 24 *μ*g/ml, fibrinogen 73 mg/dl, TAT 58 ng/ml, and PIC 17.6 *μ*g/ml. Following PRBC (4 units) infusion, he was treated with subcutaneous heparin calcium (5,000 units twice daily) and oral tranexamic acid (1,500 mg/day). After 1 week, his laboratory data improved, with Hb 9.1 g/dl, platelet count 146,000/*μ*l, FDP 10.9 *μ*g/ml, D-dimer 6.3 *μ*g/ml, and fibrinogen 186 mg/dl. Later, he was successfully switched to oral rivaroxaban (15 mg/day) as maintenance treatment at the outpatient clinic (Figure 3).

Patient 3 was a 91-year-old woman, who was hospitalized for gingival bleeding. Her laboratory data showed Hb 8.4 g/dl, platelet count 100,000/*μ*l, FDP 109 *μ*g/ml, D-dimer 51.4 *μ*g/ml, and fibrinogen 72 mg/dl. Enhanced CT revealed bilateral iliac aneurysms, with the right and left aneurysms having maximum diameters of 60.5 mm and 43.7 mm, respectively (Figure 1(c)). She was initially treated with PRBC (6 units) and fresh frozen plasma (FFP; 10 units), followed by intravenous tranexamic acid (250 mg four times daily) for 3 days. However, because these aneurysms were thought to be responsible for her coagulopathy and the patient was regarded eligible for surgery, EVAR operation was performed, after which her DIC resolved.

Patient 4 was an 83-year-old woman, who was hospitalized with gastrointestinal (GI) bleeding and dyspnea due to persistent chronic obstructive pulmonary disease. Her laboratory data showed Hb 3.0 g/dl, platelet count 62,000/*μ*l, mean corpuscular volume 82.9 (reference; 83–100) fl, serum blood urea nitrogen 98.0 (reference; 7.8–18.9) mg/dl, and creatinine 1.41 (reference; 0.45–0.82) mg/dl. Upper GI endoscopy showed bleeding in the duodenum which continued after admission, and it was found to be DIC-related. Her plasma FDP was 177 *µ*g/ml, D-dimer 81.7 *µ*g/ml, TAT 69.2 ng/ml, and PIC 12.6 *µ*g/ml. Eventually, she was identified with an aortic aneurysm (Figure 1(d)), and hemostasis laboratory abnormalities were attributed to be related to her aortic aneurysm. Surgical treatment of her aortic aneurysm was discussed; however, her general condition was poor, and because of repeat GI bleedings, she required upper GI endoscopy 13 times over 23 days for emergency hemostasis and with incomplete and persistent DIC. During the period, she received PRBC (36 units), FFP (70 units), and PC (50 units) transfusions. On day 38, she was started on systemic treatment for DIC, consisting of intravenous heparin (12,000 U/day) and intravenous tranexamic acid (250 mg twice daily), which resulted in rapid improvement of laboratory data. Unfortunately, the patient died of aspiration pneumonia 2 days later.

## 2. Discussion

Chronic DIC associated with aortic aneurysm/dissection is a primary cause of hemorrhagic diathesis in elderly patients. In aortic aneurysms, endothelial disruption associated with either dissection or atheromatous plaque rupture leads to the exposure of collagen and tissue factor, triggering the coagulation cascade, including the activation of FX, and leading to excess consumption of clotting factors and the induction of DIC [13]. Similarly, in aortic dissection, chronic expansion of the false lumen activates extrinsic tissue factors and intrinsic coagulation factors, leading to the exposure of subendothelial collagen tissue and the pooling of a high volume of blood in the false lumen. This pooled blood forms a thrombus, resulting in the continuous consumption of numerous coagulation factors [12]. As a result, DIC caused by aortic aneurysm/dissection is often classified as enhanced fibrinolytic type. DIC in these patients may therefore be controllable by inhibiting fibrinolysis.

Although the mechanism of DIC in aortic aneurysm/dissection mimics that of giant hemangioma, the incidence of clinically overt DIC ranges from 0.5% to 5.7% in patients with large aortic aneurysms compared with 25% among patients with giant hemangiomas [5, 16]. Some patients with aortic aneurysm-/dissection-related chronic DIC develop hemorrhagic diathesis, first detected when trauma or an invasive procedure such as tooth extraction triggers a sudden difficulty in achieving hemostasis. However, without such triggers, these patients may be asymptomatic, and chronic DIC may go unnoticed despite coagulation-fibrinolytic activation, in part because of compensatory mechanisms resulting in mild or even occult clinical symptoms [6, 7]. When bleeding was first identified in our four patients, two (Patients 1 and 2) had already been diagnosed with aortic aneurysm/dissection, whereas in the other two (Patients 3 and 4), aortic aneurysm/dissection was identified after bleeding developed. DIC control was immediately possible in Patients 1 and 2, delayed slightly in Patient 3, and delayed significantly in Patient 4 because of emergency care of her profuse GI bleedings in association of her poor general condition.

Hemorrhagic diathesis due to chronic DIC caused by aortic aneurysm/dissection may be corrected by surgical intervention (e.g., replacement surgery and stent grafting) or medical treatment. When possible, surgical or interventional radiology correction of the aneurysm is the primary treatment; however, this study focused on the medical treatment of chronic DIC, which requires long-term management. Three systemic therapeutic strategies for aortic DIC are available: anticoagulation therapy with heparin [8–10], antifibrinolytic therapy [11], and a combination of both [12]. The main purpose of treatment is to halt the consumption of systemic coagulation factors, fibrinogen, and platelets, as well as stop bleeding by blocking the cycle of thrombus formation and destruction. These treatments have opposite effects, preventing while generating thrombi. To date, heparin [8–10], camostat or nafamostat mesilate [17, 18], tranexamic acid [11], combinations of these medications [12], as well as rivaroxaban, a direct oral anticoagulant (DOAC) [13–15], have been reported as beneficial or successful, mostly in case reports and case series. Warfarin, on the contrary, may be less effective because it may worsen coagulopathy by reducing the expression of protein C and protein S (a cofactor of protein C) in patients with chronic DIC associated with aneurysm [14]. Stopping warfarin improved DIC parameters in Patient 1, perhaps for the reason.

In practice, patients with DIC showing severe anemia (Hb < 7.0 g/dl), low fibrinogen (<100 mg/dl), and low platelet counts (<20,000/*µ*l) should initially receive transfusions of PRBC, FFP, and PC. At the same time, patients should be started on IV or SC heparin, and its favorable pharmacokinetic profile and low cost make this an attractive option. Combinations of heparin and tranexamic acid may benefit patients with high fibrinolytic activity [12]. However, the necessity of long-term management beyond hospital stay makes it difficult to continue IV/SC heparin administration. Oral camostat mesilate [17, 18] or rivaroxaban [13–15] may be a good alternative in these patients. Rivaroxaban may suppress DIC progression by inhibiting FXa [13–15]. Patients 1 and 2 were first successfully treated with heparin (IV and/or SC) plus tranexamic acid (IV or PO), followed by oral rivaroxaban after DIC stabilized. Especially, rivaroxaban was employed as maintenance therapy to facilitate discharge from the hospital. Although administration of rivaroxaban was bolstered by several recent case reports in similar contexts [11–15], the drug has not been adapted to DIC. Therefore, its off-label use was approved by the Institutional Review Boards of our hospital in accordance with the Declaration of Helsinki (UTMC approval number #2018–20). DOACs are currently adapted for thrombotic event prophylaxis in cases of nonvalvular atrial fibrillation as well as for treatment of deep vein thrombosis-pulmonary thromboembolism, but not for treatment of aortic thromboembolism which involves the aortic aneurysm-/dissection-related chronic DIC. Considering its effectiveness, it is hoped that DOACs will be adapted for the treatment of aortic thromboembolism in future. In addition, it remains to be confirmed if other DOAC agents, such as edoxaban or apixaban, will also show similar benefits like rivaroxaban in this clinical context.

In summary, elderly patients with hemorrhagic diathesis and unexplained DIC should be assessed for aortic aneurysm/dissection. Patients diagnosed with aortic aneurysm-/dissection-related DIC should be consulted first by the vascular surgeon or interventional radiologist and be considered for systemic medical treatments. The choice of treatment depends on various factors, including underlying diseases, severity of DIC, and patient comorbidities. Nonsurgical management of chronic DIC may require a long treatment period, with use of sufficiently tolerable and effective combination treatments to maintain patients' daily activities, even outside the hospital.

## Figures and Tables

**Table 1:** Clinical features of 4 cases of chronic DIC in association with aortic aneurysm/dissection.

	Case 1	Case 2	Case 3	Case 4
Age/sex	84/F	87/M	91/F	83/F
Causes of DIC	AA	AD	CIAA/IIAA	AA
Surgical procedures (EVAR)	Yes (pre-)	Yes (pre-)	Yes (post-)	None
Site(s)/event(s) of bleeding	Subcutaneous	Tooth extraction	Gingiva	GI tract
Data on DIC				
Platelet counts (/*µ*L)	64,000	73,000	100,000	62,000
Fibrinogen (mg/dL)	98	73	72	79
FDP/D-dimer (*µ*g/mL)	101/49.8	96.8/24	109/51.4	177/81.7
TAT/PIC (ng, *µ*g/mL)	40.5/12.7	58/17.6	49/NA	69.2/12.6
DIC score^*∗*^	11	7	6	7
Treatment				
Supportive	None	PRBC	PRBC, FFP	PRBC, FFP, PC
Treatment for coagulopathy				
Initial	Heparin (DIV) + TA (DIV)	Heparin (SC) + TA (PO)	TA (DIV)	Heparin (DIV) + TA(DIV)
Later	Heparin (SC) + TA (PO)	Heparin (SC) + TA (PO)	Surgical (EVAR)	
Maintenance	Rivaroxaban (PO)	Rivaroxaban (PO)		
Outcome				
DIC	Well controlled	Well controlled	Well controlled	Delayed control
Alive/dead	Alive	Alive	Alive	Dead^*∗∗*^

DIC = disseminated intravascular coagulation, AA = aortic aneurysm, AD = aortic dissection, FDP = fibrin degradation product, TAT = thrombin-antithrombin complex, PIC = plasmin-*α*2 plasmin inhibitor complex, GI = gastrointestinal, PRBC = packed red blood cell, FFP = fresh frozen plasma, PC = platelet concentrates, TA = tranexamic acid, EVAR = endovascular aortic repair, pre- = prior to DIC development, post- = after DIC development, DIV = drip infusion, SC = subcutaneous injection, PO = per oral, CIAA = common iliac artery aneurysm, and IIAA = internal iliac artery aneurysm. ^*∗*^Based on the DIC criteria of the Ministry of Health and Welfare of Japan; ^*∗∗*^died of aspiration pneumonia.
